# The Mitochondrion-lysosome Axis in Adaptive and Innate Immunity: Effect of Lupus Regulator Peptide P140 on Mitochondria Autophagy and NETosis

**DOI:** 10.3389/fimmu.2018.02158

**Published:** 2018-09-26

**Authors:** Mykolas Bendorius, Indira Neeli, Fengjuan Wang, Srinivasa Reddy Bonam, Eszter Dombi, Nelly Buron, Annie Borgne-Sanchez, Joanna Poulton, Marko Radic, Sylviane Muller

**Affiliations:** ^1^Unit Biotechnology and Cell Signaling, Laboratory of Excellence Medalis, CNRS, Strasbourg University, Illkirch, France; ^2^Department of Microbiology, Immunology and Biochemistry, University of Tennessee Health Science Center, Memphis, TN, United States; ^3^Nuffield Department of Women's and Reproductive Health, Women's Centre, Oxford, United Kingdom; ^4^Mitologics SAS, Paris, France; ^5^Institute for Advanced Study, University of Strasbourg, Strasbourg, France

**Keywords:** NETosis, autophagy, mitochondrion, systemic lupus erythematosus, neuroinflammation, P140 peptide

## Abstract

Mitochondria deserve special attention as sensors of cellular energy homeostasis and metabolic state. Moreover, mitochondria integrate intra- and extra-cellular signals to determine appropriate cellular responses that range from proliferation to cell death. In autoimmunity, as in other inflammatory chronic disorders, the metabolism of immune cells may be extensively remodeled, perturbing sensitive tolerogenic mechanisms. Here, we examine the distribution and effects of the therapeutic 21-mer peptide called P140, which shows remarkable efficacy in modulating immune responses in inflammatory settings. We measured P140 and control peptide effects on isolated mitochondria, the distribution of peptides in live cells, and their influence on the levels of key autophagy regulators. Our data indicate that while P140 targets macro- and chaperone-mediated autophagy processes, it has little effect, if any, on mitochondrial autophagy. Remarkably, however, it suppresses NET release from neutrophils exposed to immobilized NET-anti-DNA IgG complexes. Together, our results suggest that in the mitochondrion-lysosome axis, a likely driver of NETosis and inflammation, the P140 peptide does not operate by affecting mitochondria directly.

## Introduction

Mitochondria are specialized cytoplasmic organelles known to generate cellular energy, converting oxygen, and nutrients into adenosine triphosphate (ATP), that are now emerging as true metabolic sensors. Mitochondria are present in all nucleated cells and in platelets where they affect a wide array of vital cell functions, notably in cellular stress responses such as autophagy and apoptosis. They exert crucial roles in reactive oxygen species (ROS) signaling, which is important in hypoxia sensing, and in cellular differentiation during development (1–3). Mitochondria are also central to innate immunity (4).

Disruption of the mitochondrial genetic material or mitochondrial metabolic functions contributes to numerous pathologies. Even though primary genetic mitochondrial myopathies remain relatively rare, the prevalence of disorders due to secondary mitochondrial dysfunction (caused by pathological events originated outside mitochondria) is much higher. Defective mitochondrial functioning contributes to cardiac diseases (ischemia/reperfusion injury), metabolic syndrome (obesity), and neurodegenerative diseases, such as Huntington's, Parkinson's, and Alzheimer's diseases (5). Cells of the central nervous system and muscles impose high demands on energy supply and are therefore particularly susceptible to mitochondrial insufficiency. In systemic lupus erythematosus (SLE), a chronic inflammatory autoimmune syndrome, mitochondrial respiration is critical for neutrophil extracellular trap (NET) formation, and mitochondria released by neutrophils induce inflammatory cytokine production (6). Mitochondrial ROS inhibition was thus found to reduce disease severity and type I interferon responses in a mouse model of lupus (6). Although autoantibodies (autoAb) to citrullinated proteins suggest that NETosis makes an important contribution to autoimmunity, experiments in mice have led to inconsistent results. For example, the manifestations of autoimmunity are more severe in mice with deficiencies that are expected to reduce the ability of neutrophils to release NETs (7, 8). One possible explanation for the discordant results is the fact that mice and humans respond differently to citrullinated autoantigens (9).

NETosis, named so because it involves the release of NETs as part of a regulated, multi-step cell death characteristic of neutrophils, plays an important role in autoimmune diseases. In SLE, NETs released by activated neutrophils form lattices of decondensed chromatin fibers containing intact DNA filaments, histones, and neutrophil enzymes. Each of these macromolecules generate prominent (auto)immune Ab targets (10–13). Autoreactivity of NET components is enhanced by post-translational modifications, such as deimination of arginine residues (11, 12) in histones and other neutrophil proteins that enhance their immunogenicity (14, 15). Curiously, autophagy, a complex genetically-regulated mechanism involved in the cell survival/death balance, affects NETosis. It determines the efficiency of neutrophils for NET release, including in inflammatory disorders (16) and infectious diseases (17, 18). Despite clear evidence from different experimental systems, the precise autophagy regulators required for the execution of NETosis is somewhat controversial (19, 20).

Autophagy is a tightly regulated mechanism that allows cells to renew themselves through the lysosomal degradation of damaged organelles and of proteins, which are misfolded or produced in excess. It is functionally central in many compartments of the cell as it maintains homeostasis and plays important roles in the immune response that may be connected to NET formation (21–24). There are three major pathways that characterize bulk autophagy, namely macroautophagy, chaperone-mediated autophagy (CMA), and microautophagy. Other forms of autophagy exist that are more specific, for example mitophagy, which involves the selective degradation of mitochondria, lipophagy that results in degradation of lipids, and xenophagy, in elimination of invading pathogens. Recent publications describe multiple effects of autophagy defects in different autoimmune and inflammatory processes (25–30). At this stage, however, the data are still scarce and incomplete, and the molecular and cellular links between NETosis and autophagy remain largely unexplored (31–34). This question is central as inhibition of autophagy and ROS might prevent intracellular chromatin decondensation, a key step of NETosis.

To explore the relationships of NETosis, autophagy and mitochondria, we employed several biochemical and cellular approaches with both human cell lines and primary cells from patients. We exploited a peptide called P140 known to be rapidly endocytosed *via* a clathrin-mediated endocytosis (CME) pathway and ultimately accumulated into lysosomes of B cells after its intravenous administration into MRL/lpr lupus-prone mice (35). P140, a 21-mer phosphopeptide derived from the spliceosomal protein U1-70K, was found to act directly on CMA, which appears to be hyperactivated in MRL/lpr splenic B cells (35), and most likely indirectly, on the macroautophagy process (36–38), which also shows higher activity in both T and B cells in murine lupus (39–42). As a close functional link between lysosomes (where CMA is active) and mitochondria has been suggested (3, 43, 44), it was important to first examine whether P140 peptide could alter functions of mitochondria and modulate mitophagy. Our data show that P140 inhibits some of mitochondrial properties but has no effect on mitophagy, indicating the selectivity of P140 peptide for CMA (and macroautophagy). Regarding the effect of P140 on NETosis, we did observe that P140 suppresses NET release from neutrophils stimulated with autoimmune NET-IC (NICs). Thus, in an autoimmune context, P140 could decrease NET release and dampen the exposure of nuclear autoantigens, therefore attenuating immune responses to self-antigens, an observation we made previously both in mice and patients with lupus. Our results, therefore, highlight the importance of the mitochondrion-independent pathway in NETosis, which seems to be more specially modulated by P140. The exact target of P140 in this pathway remains to be identified.

## Materials and methods

### Peptides

The P140 (RIHMVYSKRpSGKPRGYAFIEY), scrambled (Sc) P140 (YVSRYFGpSAIRHEPKMKIYRG) phosphopeptides (pS standing for phosphoserine residues), and non-phosphorylated peptide 131–151 (RIHMVYSKRSGKPRGYAFIEY) were synthesized as described previously (45). Peptides homogeneity was checked by analytical high-performance liquid chromatography and their identity was assessed by mass spectrometry.

### Live imaging analysis by spinning disk confocal microscopy

MRL/N-1 fibroblastoid cells established from the spleens of MRL/MpTn-*gld/gld* mice (46) were incubated with 10 μM AF633-P140 for 4 h followed by staining with 100 nM LysoTracker Green at 37°C for 5 min, or cells were incubated with 10 μM AF488-P140 for 4 h followed by staining with 50 nM MitoTracker DeepRed at 37°C for 20 min. Stained cells were washed three times with phosphate-buffered saline (PBS) pH 7.4, and imaged immediately with a spinning-disc confocal microscope consisting of a CSU confocal spinning disk unit (Yokogawa), an EMCCD Evolve camera (Roper Scientific), mounted on an Axio Observer Z1 microscope (Zeiss) at 37°C with 5% CO_2_ supply. Both LysoTracker Green and MitoTracker DeepRed were purchased from ThermoFisher Scientific.

### Primary fibroblast cultures

For analyzing the effect of P140 on mitophagy, primary fibroblasts established from skin biopsies obtained from two symptomatic patients harboring different loads (both <40%) of the m.3243A > G “MELAS” (mitochondrial encephalopathy with lactic acidosis and stroke episodes) mutation (47) were tested with informed consent of patients and the approval from the UK National Research Ethics Service. These two patients are identified here as P1 and P2. MELAS is a rare progressive multisystemic disorder that particularly affects the brain and nervous system (associated to neurological and psychiatric manifestations), and muscles, with onset typically in childhood. Patients also develop endocrinopathy, heart disease, diabetes, and hearing loss. We also used a control from a panel of 22 anonymized control fibroblast cultures established either with parental consent from children undergoing diagnostic skin biopsy for karyotyping and whose cytogenetic markers were normal (*n* = 10) or from healthy consented adults aged 18–81 years (*n* = 12). The control used was close to the median for all functional tests carried out.

### Effect of P140 on mitochondria purified from Raji cells

Raji B cells were cultured in Roswell Park Memorial Institute (RPMI) 1640 medium (Gibco) supplemented with 10% (v/v) fetal calf serum (FCS), 4-(2-hydroxyethyl)-1-piperazineethanesulfonic acid (HEPES), and gentamycin. The medium was changed every 2–3 days and cells were split when reaching 2-3 × 10^6^ viable cells/mL. The experiments were done using a cell density of 1 × 10^6^ Raji B cells/mL. Cell survival was measured using the 3-(4,5-dimethylthiazol-2-yl)-5-(3-carboxymethoxyphenyl)-2-(4-sulfophenyl)-2H-tetrazolium (MTS) cell proliferation colorimetric assay kit from Abcam. Mitochondria from Raji cells were isolated as described previously (48) and re-suspended in homogenization buffer (300 mM sucrose, 5 mM 2-[Tris(hydroxymethyl)-methylamino]-ethanesulfonic acid/TES buffer pH 7.2, 0.2 mM ethylene glycol tetraacetic acid, 1 mg/mL BSA). Mitochondrial swelling and ΔΨm were evaluated as described (48, 49) in the presence of succinate and rotenone. Mitochondria were incubated with or without P140 or ScP140 peptides during 5 min before absorbance at 545 nm (swelling) and Rh123 fluorescence (ΔΨm, λ_Excitation_ 485 nm, λ_Emission_ 535 nm) recording during 45 min using a fluorescence multi-well plate reader (Infinite, Tecan®). Calcium (CaCl_2_; 50 μM) and carbonyl cyanide 3-chlorophenylhydrazone (50 μM) were used as the 100% baseline for swelling and ΔΨm loss respectively. Oxygen consumption was monitored as described (49) in the presence of the O_2_ sensitive dye MitoXpress® (LUXCEL). Mitochondria were incubated with or without P140 or ScP140 during 5 min before the measure of oxygen consumption during 45 min in 96-well plates using a spectrofluorimeter (Infinite® 200; λ_Excitation_ 380 nm, λ_Emission_ 650 nm). Rotenone (2 μM) and oligomycin A (1 μM) were used as 0% baseline for oxygen consumption driven by complex I and complex II respectively. The areas under curve were used for calculations. For measuring ATP production, 2 μg of isolated mitochondria were pre-incubated with or without P140 or ScP140 during 5 min in the presence of 25 mM succinate, 2 μM rotenone and 1.65 mM ADP in oxygen consumption buffer (49) ATP produced during this time was monitored using the ApoSENSOR kit (BioVision) by spectrofluorimeter (Infinite® 200; luminescence detection). Results were expressed in percent of ATP production after normalization by positive (untreated cells; 100%) and negative (25 mM malonate; 0%) controls. For studying mitochondrial localization, 20 μg of isolated mitochondria were incubated with or without AF488-peptides P140 or ScP140 during 5 min at 37°C before washes with homogenization buffer at pH 11.6 and centrifugation at 10,000 g for 10 min. The pellet was next resuspended in homogenization buffer at pH 7.2 before analysis by flow cytometry (FACSCalibur; λ_Excitation_ 488 nm, λ_Emission_ 530 nm).

### Evaluation of mitophagy

Primary fibroblast from control individuals and patients (see above) were seeded in a 96-well plate at a density of 2,500 cells/well. Next day the media was changed for regular Dulbecco's Modified Eagle's medium (DMEM) containing 25 mM glucose or glucose-free galactose media (0 mM glucose, 10 mM galactose). P140 and control ScP140 peptide were given to cells at a concentration of 40 μM for 30 min, 2, 6, 16, and 24 h before the cells were fixed with 4% (v/v) paraformaldehyde (PFA) for 15 min at room temperature (RT). The cells were then immuno-stained using Abs raised against transporter outer membrane (TOM) 20 (mouse, 1/200; Santa Cruz, sc6341) and microtubule-associated protein 1A/1B-light chain 3B (MAP1LC3B; rabbit, 1/500; Caltag Medsystems, PM036) and revealed with secondary Abs anti-mouse AF546 and anti-rabbit AF488, diluted 1/200 and 1/400, respectively. Nuclei were stained using 4′,6-diamidino-2-phenylindole (DAPI). Cells were then imaged using the IN Cell 1000 analyzer (GE Healthcare), 9 fields of view per well and the images analyzed using a development of a specialized Developer toolbox protocol (50). For the mitochondrial DNA experiments, the fixation step was replaced by a staining using PicoGreen solution (Molecular Probes Inc.) diluted at 3 μL/mL directly into cell culture medium and tetramethyl rhodamine methyl ester, a cell-permeant, cationic, red-orange fluorescent dye (25 nM final concentration), for 45 min in an incubator. The media was washed away and replaced with fresh media and the cells imaged and analyzed using the IN Cell 1000 analyzer and Developer toolbox.

### Evaluation of autophagy processes in neuronal cells

Adherent U-251 MG cells (ECACC, ref. 0906300) were selected for this study as a model. Cells were maintained in DMEM (ThermoFisher, ref. 41965-039), containing 10% (v/v) FCS (ref. 26140-079), 100 units/mL of penicillin and 100 μg/mL of streptomycin (ref. 15140-122) all from ThermoFisher. Cells were subcultured after reaching 70–80% confluency and their doubling time was 22 h (51). For studying the effect of P140 and ScP140 peptides on autophagy levels, cells were seeded at 0.2 × 10^6^ cells per 12-well plate well and incubated for 16 h at 37°C. They were treated or not for 8 h with 10 μM P140. To measure the autophagic flux, half of P140-samples and controls were incubated with 5 μg/mL of each pepstatin A, a potent inhibitor of aspartyl proteases (Merck, 5318) and E-64d/Aloxistatin, a pan-cysteine cathepsin inhibitor (Merck, E8640). Cells were lyzed in RIPA buffer, pH 7.6 (ThermoFisher, ref. 89900), transferred in Laemmli buffer for analysis on SDS-PAGE, and analyzed by western immunoblotting (30). Ten μg protein was loaded per lane as quantified by bicinchoninic acid assay. The conditions used to measure autophagy markers were as described (30) using Abs to MAP1LC3 (MBL, M186-3; 0.5 μg/mL), sequestosome-1/p62 (SQSTM1; Abcam, ab109012; 0.5 μg/mL), B-cell lymphoma (BCL)-2 interacting myosin/moesin-like coiled-coil protein 1 (BECLIN-1; Abcam, ab207612; 0.5 μg/mL), autophagy-related protein (ATG) 12/5 (Abcam, ab155589; 1 μg/mL), lysosome-associated membrane protein-2A (LAMP2A; Abcam, ab18528; 1 μg/mL) and phosphatase and tensin homolog deleted on chromosome 10 (PTEN)-induced putative kinase 1 (PINK1; Abcam, ab75487; 1 μg/mL). To avoid quantification mistakes resulting from the fact that a loading control protein could represent a substrate for autophagy, normalization of blots was done using stain-free technology (total protein lane content).

### Isolation of human peripheral blood neutrophils

Neutrophils were isolated from heparinized blood from healthy donors, in accord with protocols approved by the University of Tennessee Institutional Review Board, and isolated following published methods (52). Briefly, neutrophils were purified at RT, enriched by Isolymph gravity sedimentation, and recovered in the pellet of an Isolymph density gradient (CTL Scientific Supplies; ref. 759050) under endotoxin-free conditions. The contaminating erythrocytes were lysed in ice-cold hypotonic (0.2%, w/v) sodium chloride solution for 30 s, at which point the solution was rendered physiologic saline by addition of hypertonic (1.6%, w/v) sodium chloride. The neutrophils were rinsed once in Hanks' balanced salt solution (HBSS; without calcium or magnesium and with 10 mM HEPES) and re-suspended at 2 × 10^6^ cells/mL in the same buffer. Neutrophil viability was found to be 98% by Trypan blue dye exclusion.

### Preparation of immobilized immune complexes (ICS)

Plate-bound ICs were prepared by coating wells of 96-well black tissue culture plates (ThermoFischer; ref. 165305) with purified monoclonal Ab 3H9 at 5 μg/mL in PBS overnight at 4°C. The 3H9 mouse monoclonal IgG was grown and purified from culture supernatants by Protein A beads, as described previously (52). The next day, plates were washed once with PBS and blocked with 2% (w/v) IgG- and protease-free BSA (Jackson ImmunoResearch Labs; ref. 001-000-161) and 10 μg/mL poly-L-Lysine in PBS for 1 h at RT, followed by 2 washes with PBS. NETs, prepared from healthy human neutrophils by incubation with hydroxyapatite and isolated according to previously described procedures (53), were added to the wells at a concentration of 2 μg/mL protein and 4 μg/mL DNA. Following incubation for 1 h at RT, the plates were washed 3 times with HBSS.

### Effect of P140 on NETS release from human neutrophils

Freshly isolated human neutrophils were incubated for 15 min at RT with different concentrations of P140 or control peptides in HBSS without Ca/Mg but containing 10 mM HEPES. Neutrophil-peptide suspensions were then transferred to wells of IC-coated plates, and cells were allowed to settle at the bottom of the wells for 10 min at 37°C in a CO_2_ incubator. Fifty μL of HBSS with HEPES and Ca/Mg were added to the wells. NET release was measured by fluorescence of 2.5 μM cell-impermeable Sytox Green (Molecular Probes; ref. S7020) in a Synergy plate reader (BioTek Instruments, set for excitation at 488 nm and emission at 510 nm).

### Statistical analysis

Statistical analyses were performed using GraphPad Prism version 5.0. Depending on the number of samples that were included in the analyses, and the distribution of data, statistical significances were assessed using the parametric, Student's *t*-test or the non-parametric Mann-Whitney's test. For comparing the significance of data obtained with P140 and ScP140, *P* values were determined by using one-way ANOVA. *P* < 0.05 were considered significant.

## Results

### Effect of P140 peptide on isolated mitochondria

Mitophagy is essential for the degradation of damaged mitochondria and therefore occurs constantly at a basal level. Mitochondrial dysfunction is known in lupus, notably in patient's T cells (persistent mitochondrial hyperpolarization, cytoplasmic alkalization, increased reactive oxygen intermediates production, and diminished levels of intracellular glutathione and ATP) (54–56). Although the mammalian target of rapamycin (mTOR), a sensor of mitochondrial homeostasis in T cells, is activated in SLE patients, blockade of mTOR with rapamycin, for example, incompletely reverses mitochondrial hyperpolarization and fails to correct accumulation of mitochondria, suggesting that mitochondrial dysfunction occurs upstream of mTOR activation in SLE (57). In this context, we therefore performed a series of experiments to determine whether P140 peptide interacts with purified mitochondria and if it could have some effect on mitophagy, in parallel to its effects on lysosomes and CMA/macroautophagy (35, 36, 38).

**Figure 1:**
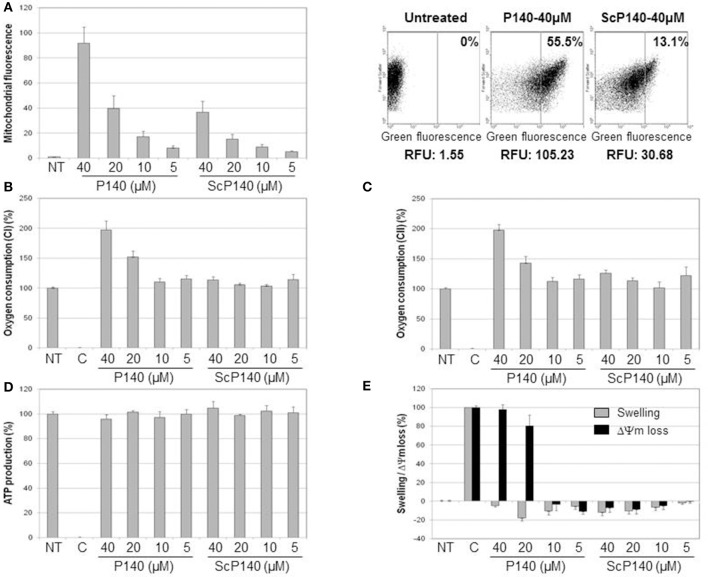
Direct effect of P140 peptide on mitochondria isolated from Raji cell line. **(A)** Mitochondrial targeting was estimated by flow cytometry after incubation of increasing concentrations of AF488-P140 and ScP140 peptides with isolated mitochondria. Left panel: mitochondrial labeling was expressed in fluorescence ratio in function of mitochondrial basal fluorescence (Untreated; mitochondria without peptide). Right panel: relative mitochondrial fluorescence with or without incubation with AF488-peptides. The percentage of highly labeled mitochondria (right section) and relative fluorescence unit were indicated for each sample. **(B,C)** Oxygen consumption in the presence of malate, glutamate and ADP **(B)** or succinate **(C)** was measured with or without increasing concentrations of P140 and ScP140 peptides. Results were normalized on both untreated mitochondria (100% of oxygen consumption) and inhibition control (rotenone for complex I and oligomycin A for complex II; 0% of oxygen consumption). **(D)** ATP production of mitochondria in the presence of succinate and ADP was measured with or without increasing doses of P140 and ScP140 peptides. Results were normalized on both untreated mitochondria (100% of ATP production) and inhibition control (malonate; 0% of ATP production). **(E)** Mitochondrial swelling (gray bars) and potential loss (ΔΨm loss; black bars) were measured with or without increasing concentrations of P140 and ScP140 peptides. Results were normalized on both untreated mitochondria (0% of swelling and ΔΨm loss) and control (Triton for 100% optical density decrease and carbonyl cyanide 3-chlorophenylhydrazone for 100% ΔΨm loss).

In a dose-dependent manner, AF488-labeled P140 peptide was detectable at higher levels than the ScP140 control in mitochondria purified from Raji cells (Figure 1A). This marked rise of fluorescence was observed even after washing at pH 11.6, which removes components non-specifically bound to the mitochondrial outer membrane, leading us to conclude that the binding of P140 to mitochondria was both strong and specific. P140 (but not ScP140) also affected respiratory capacity of mitochondria. It strongly increased oxygen consumption driven by both succinate oxidation (complex II) and malate and glutamate oxidation (complex I) (Figures 1B,C). This stimulation of oxygen consumption was associated with a strong perturbation of mitochondrial potential, without any swelling and significant modification of ATP production (Figures 1D,E). P140 may therefore perturb proton and/or electron transfer through mitochondrial inner membrane, leading to potential loss and oxidative phosphorylation uncoupling, hence triggering an important stimulation of oxygen consumption, while ATP production remains stable during the time of the measure. There was no impact of P140 tested in a concentration range of 0 to 100 μM on cell survival as measured by MTS cell proliferation colorimetric assay at 72 h on Raji B-cell lymphoma line (not shown).

### Effect of P140 peptide on mitophagy

**Figure 2:**
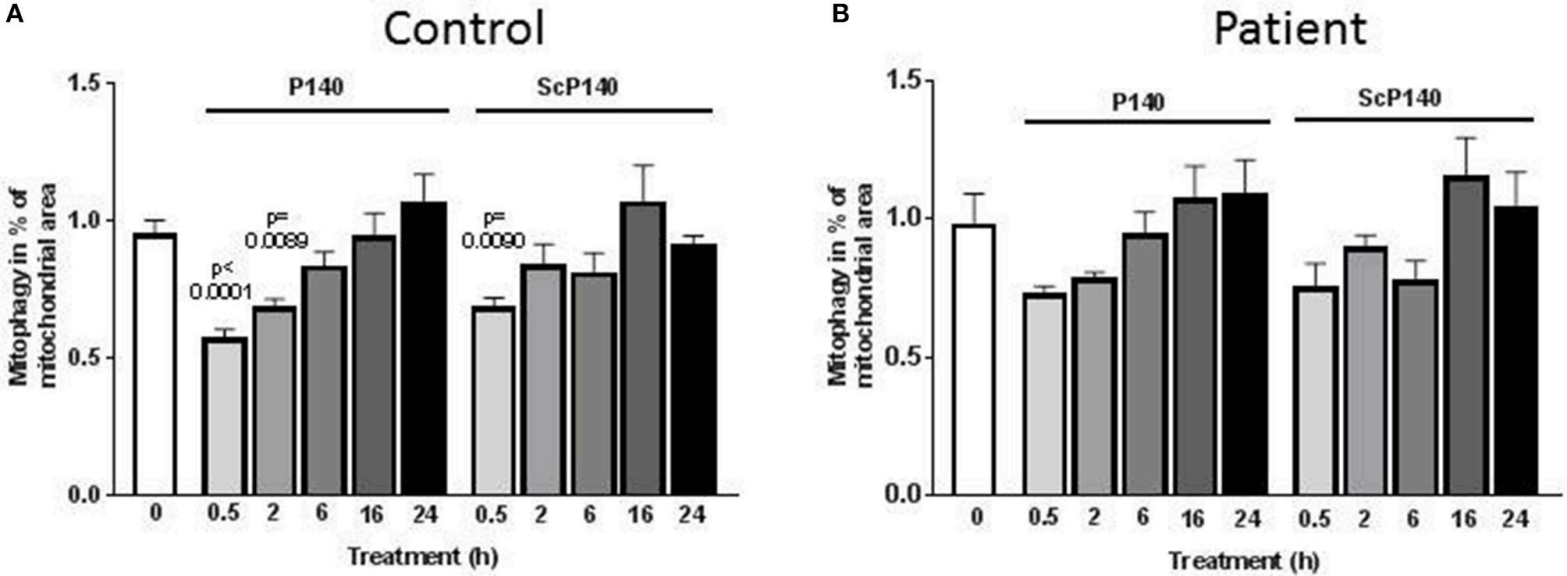
Effect of P140 on mitophagy. Control **(A)** and patient's **(B)** fibroblasts (harboring the m.3243G>A mitochondrial DNA mutation) were cultured in 96-well plates in high-glucose media. They were exposed to P140 or ScP140 peptides (40 μM final concentration) for 30 min to 24 h. MAP1LC3-positive puncta counts were evaluated by imaging cells using the INCell 100 analyzer. Mitophagy was determined as the co-localization of mitochondria (stained with anti-TOM20 Ab) and autophagosomes (stained with anti-MAP1LC3 Ab). A minimum of 275 cells were analyzed per condition. Error bars are standard error of the mean (SEM). *P* values are expressed vs. the time 0.

We then sought to assess the effect of P140 on mitophagy. This was done using a high-throughput fluorescence microscopy quantitative assay (IN Cell 1000), which was developed to screen drugs that might modulate mitophagy (50). Because baseline autophagy and mitophagy are increased in cells with mitochondrial dysfunction, we used skin fibroblasts from one control and two patients suffering from MELAS [rare syndrome associated with mitochondrial encephalomyoapthy and psychiatric disorders (47)], one who was mildly affected and the other who was significantly sick. Similar autophagosome counts were registered in the presence of P140 and ScP140 (Figure 2; Supplementary Figures S1–S3), leading to the conclusion that P140 peptide displays no specific effect on mitophagy. We also excluded a significant P140 effect on mitophagy, using co-localization of mitochondrial fragments and autophagosomes, or on mitochondrial length.

Taken together, our results support the view that P140 exerts some effects on isolated mitochondria from Raji B cells by influencing transmembrane potential and oxygen consumption but has no impact on mitophagy in patient's fibroblasts.

### Effect of P140 on macroautophagy and CMA processes in neuronal cells

To extend our observation on macroautophagy and CMA of P140 in neuronal cells, we selected U-251 MG cells, formerly known as U-373 MG, as a model. This human astrocytoma cell line was initially derived from a malignant glioblastoma tumor by explant technique. These cells are supposed to display some immune functions, notably as antigen-presenting cells, and continuously express glial fibrillary acidic protein, a prototypical marker of astrocytes (58) and a CMA regulator (59). Astrocytes produce a wide range of pro- and anti-inflammatory cytokines and seem to play complex roles in autoimmune inflammatory disorders, e.g., in multiple sclerosis and neuropsychiatric lupus (58, 60, 61). Astrocyte-expressed cytokines can exert potent suppressive effects on inflammatory cells (62).

**Figure 3:**
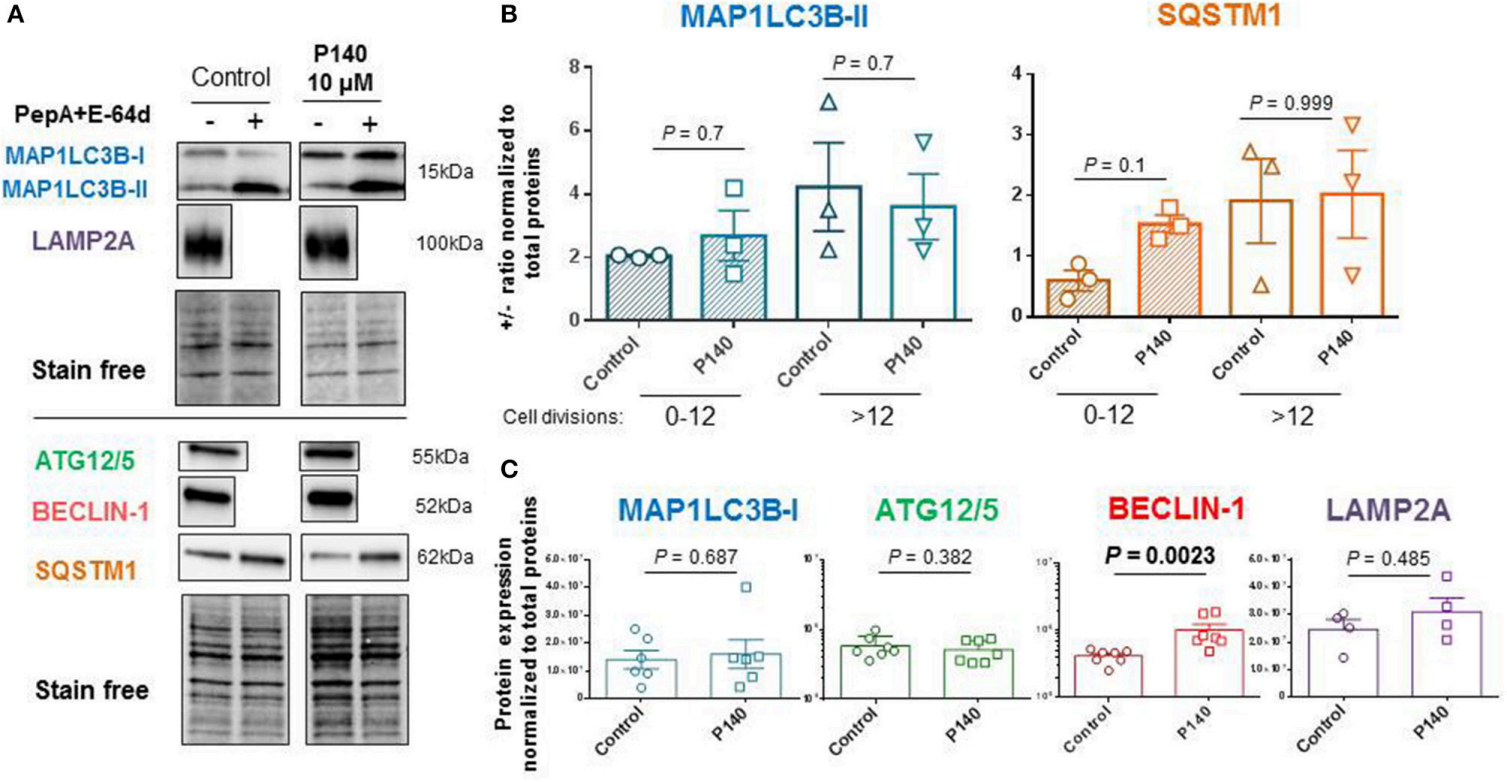
Effects of P140 on autophagy in U-251 MG glioblastoma cells. U-251 MG cells were treated or not with 10 μM P140 for 8 h in the presence or absence of lysosomal enzyme inhibitors pepstatin A (PepA) and E64D. Protein lysates were separated on 4–20% SDS-gels and then transferred onto nitrocellulose membrane. **(A)** Proteins were revealed with Abs to ATG12/5, MAP1LC3B, SQSTM1, BECLIN-1, and LAMP2A. Comparisons were made between untreated and P140-treated U-251 MG cells. Normalization was performed by measuring total protein directly on the membrane (stain-free procedure). **(B)** Autophagic markers MAP1LC3B and SQSTM1 measured in the absence and presence of anti-proteases as indicators of flux. The effect of P140 peptide on the flux intensity is shown. The results are displayed with cells collected after 1–12 cycles of divisions or after a longer period of more than 12–20 cycles of division in culture medium. **(C)** Effect of P140 on ATG12/5, BECLIN-1, and LAMP2A protein expression. Error bars are SEM. Each sample was tested in triplicates or quadruplets in at least 3 independent experiments (1 point corresponds to the mean value of replicates). *P* values are from Mann-Whitney U tests.

The autophagic flux as measured by the MAP1LC3-II expression in U-251 MG cells in the presence or absence of anti-proteases was moderate in the absence of any autophagy activator or conditions of nutrient deprivation (Figures 3A,B). It was found to depend on the number of cell divisions with a more active flux in cells generated after several division cycles compared to cells, which had a low number of divisions in culture medium (Figure 3B). In these basal conditions, and in both cases, P140 had no detectable effect on the autophagic flux. These results were confirmed by measuring the expression levels of another macroautophagy-linked protein, SQSTM1 (Figure 3B). P140 had also no effect on the accumulation of MAP1LC3-I (Figure 3C). Likewise, the expression of ATG5/12, which is involved in the extension of the phagophore membrane in autophagic vesicles, was not affected by P140 (Figure 3C). However, the expression level of BECLIN-1, which is involved in the very early stages of autophagosome formation (63) was significantly increased (*P* = 0.0023; Figure 3C).

**Figure 4:**
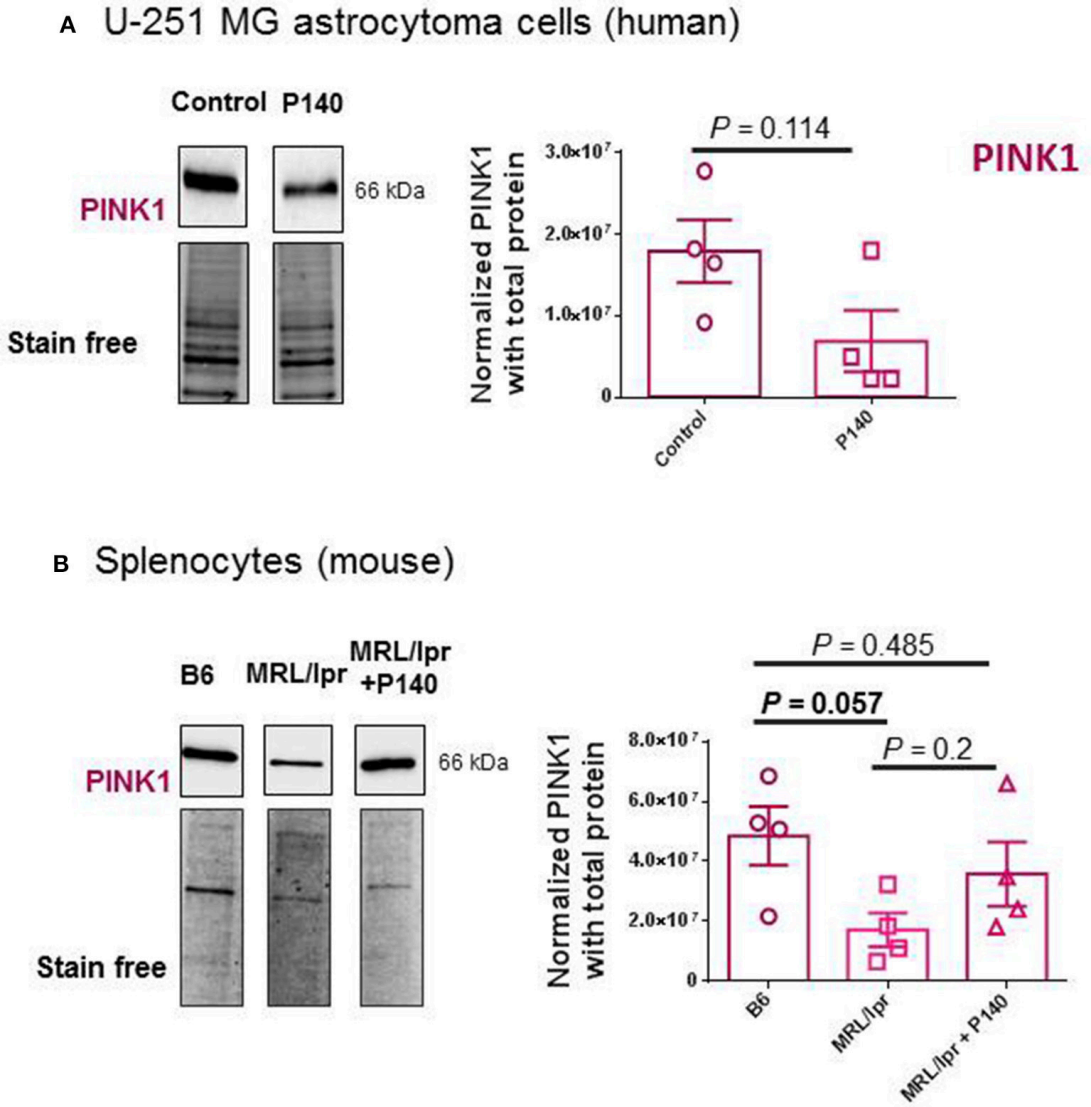
Effects of P140 on PINK1 expression. PINK1 expression was measured by western blotting in P140-treated and non-treated human U-251 MG cells **(A)** and in splenocytes collected from untreated normal B6 mice and P140-treated or non-treated MRL/lpr mice **(B)**. The experimental conditions were as in Figure 3 using PINK1 antibodies. Each sample was tested in quadruplets in 4 independent experiments. Error bars are SEM. *P* values are from Mann-Whitney U tests.

The possible effect of P140 on other forms of autophagy was also examined in U-251 MG cells. CMA activity was evaluated by measuring the expression of LAMP2A by western blot (30) and found to be unaffected by P140 (Figure 3C). The expression of the mitochondrial membrane protein PINK1 was not affected either in U-251 MG cell cultures that were treated or not by P140 peptide (Figure 4A). This absence of P140 effect was confirmed by measuring PINK1 expression in MRL/lpr splenocytes (Figure 4B).

**Figure 5:**
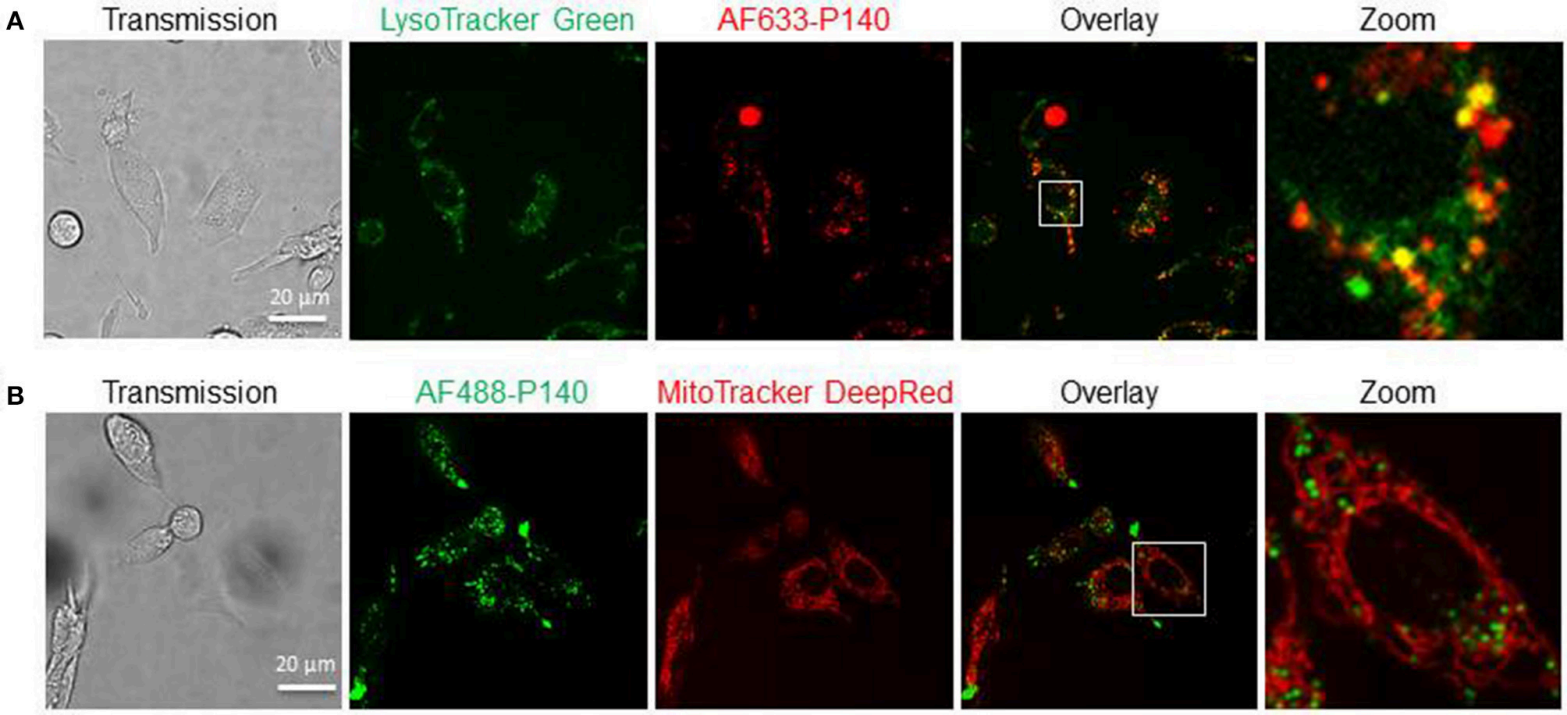
Cellular localization of P140 peptide. Confocal images of MRL/N-1 cells treated with 10 μM AF633-P140 for 4 h and stained with LysoTracker Green **(A)**, or with 10 μM AF488-P140 for 4 h and stained with Mito Tracker DeepRed **(B)**. Images are analyzed in Image J (NIH). A magnification of the images is shown in right panels (zoom).

Together these results support the view that P140 is able to affect basal macroautophagy in astrocytoma cell line U-251 MG cells, while it has no effect on mitophagy. This conclusion was reinforced by live-cell imaging experiments using laser scanning confocal microscopy and fluorescent labeled P140. Confocal images of MRL/N-1 cells treated *in vitro* with fluorescent P140 for 4 h showed that the peptide readily co-localized with lysosomal vesicles revealed by LysoTracker Green staining (Figure 5A) but not with mitochondria visualized with mitoTracker DeepRed dye (Figure 5B).

### Effect of P140 on NETosis

**Figure 6:**
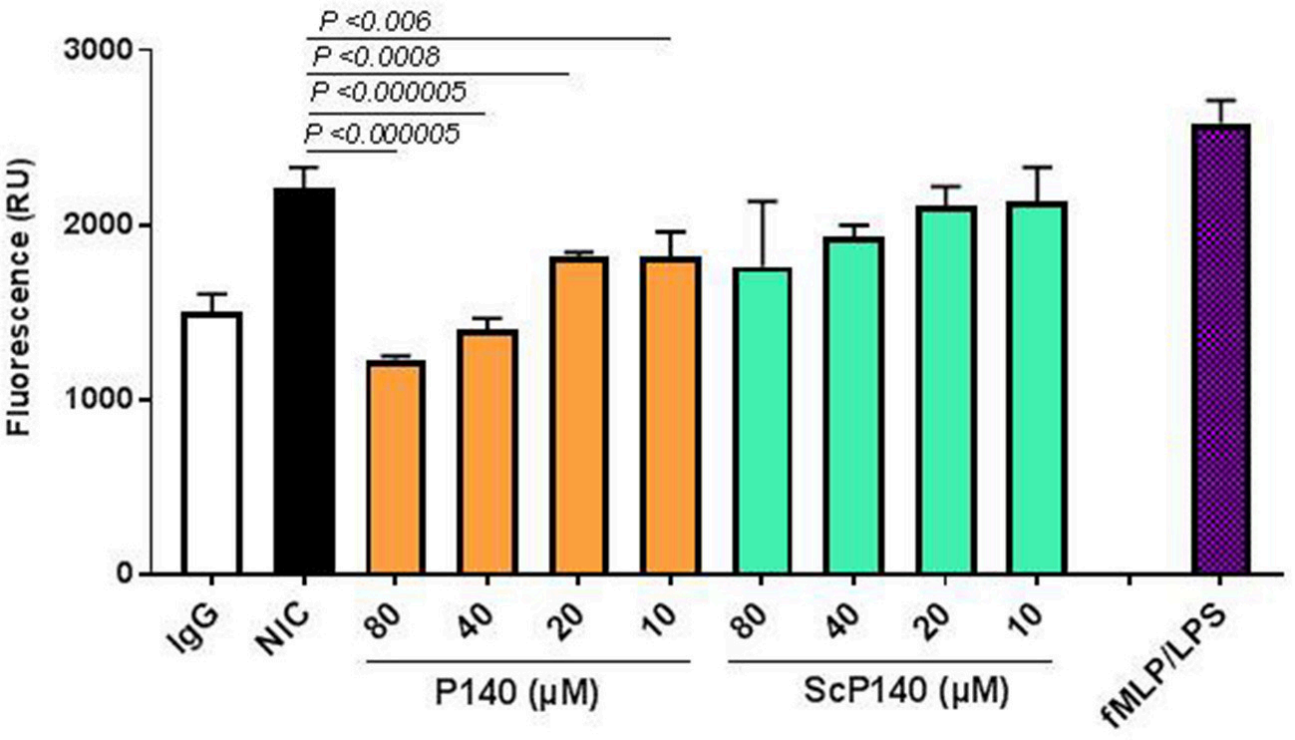
NIC-induced NET release from neutrophils incubated with P140 or control peptides. The NETs released in response to immobilized anti-DNA IgG 3H9 and purified NETs were quantified by fluorescence (expressed as relative units, RU). Readings were recorded at 120 min from neutrophils incubated without peptide, neutrophils incubated with LPS and fMLP (as positive control), and neutrophils that were incubated with increasing concentrations of P140 or ScP140. This set of experiment was performed at least five times with samples tested in triplicates in 96-well plates with consistent results. Error bars are SD. *P* values indicate probability thresholds for significant differences between NIC-incubated cells vs. cells pretreated with either P140 or control ScP140. *P* values were determined by using one-way ANOVA.

To examine the effect of P140 on NET release, we developed an assay in which healthy human neutrophils are stimulated by incubation with immune complexes formed between a murine anti-DNA monoclonal Ab, 3H9, and purified NETs. Immobilized NICs elicited NET release above the levels of 3H9 alone and the observed NET release approached levels elicited by lipopolysaccharide (LPS) and N-Formylmethionyl-leucyl-phenylalanine (fMLP), used here as internal controls (64). fMLP is a peptide that is generated by proteolysis of bacterial polypeptides and induces neutrophil chemotaxis and degranulation. Pre-incubation of neutrophils with P140 suppressed NIC-induced NET release in a dose-dependent manner, whereas the ScP140 showed no such effect over the same concentration range (Figure 6). The P140 peptide concentrations that inhibited NET release in response to NIC stimulation were ineffective as inhibitors of fMLP/LPS (not shown) or phorbol myristate acetate/ionomycin (33)-stimulated NET release under conditions used for the positive control (Figure 6).

## Discussion

Our previous studies demonstrated that following intravenous injection into lupus-prone MRL/lpr mice, P140 enters B cells through CME, and homes into lysosomes where it potentially blocks lupus-related hyperactivated CMA (35, 38). *In vitro*, P140 destabilizes the complex formed by CMA chaperones heat shock protein (HSP)A8 and HSP90, and cofactors (35). By reducing excessive CMA and macroautophagy, P140 peptide may alter antigen presentation (65) and lead to a reduced stimulation of autoreactive T cells. Reduction of MHC expression and autoreactive T cell hyporesponsiveness was effectively observed in P140-treated MRL/lpr mice and human P140-treated peripheral blood cells (36, 66–68). In the salivary glands of MRL/lpr mice, similar to what was observed in the spleen, the therapeutic CMA-regulator P140 peptide reduced the abnormally raised lysosomal pH and rescued the altered autophagy functions (69).

Our present findings show that P140 does not alter the autophagic flux measured in human astrocytoma U-251 MG cells in basal culture conditions and in the absence of any overt stress or stimulus. The expression levels of several components of the macroautophagy and CMA processes, namely MAP1LC3, SQSTM1, ATG12/5, and LAMP2A, were not affected by peptide treatment. However, the expression of BECLIN-1, a mammalian homolog of yeast Vps30/Atg6, was raised in an impressive manner. This result is important because BECLIN-1, which forms part of the class III PtdIns3K complex that also contains VPS34, VPS15, and ATG14 is involved in the initial steps of autophagosome formation and is a central regulator of autophagy (70–72). BECLIN-1 deficiency has been characterized in several pathological conditions and enhancing autophagy, for example with the Tat-BECLIN-1 construct, has been presented as a potential route for valuable therapeutic applications, notably in neurodegenerative diseases (63, 73, 74).

The results presented here further suggest that in contrast to its effect on CMA (shown in MRL/lpr primary spleen cells) and macroautophagy, P140 does not affect mitophagy, although a significant functional effect of P140 could be visualized *in vitro* when isolated mitochondria were used. This finding is corroborated by microscopy observations indicating that labeled-P140 co-localizes with lysosomes but not with mitochondria.

To explore further the effects of P140 and control peptides on innate immune cells, we developed a fluorescence assay that measures NETs released into the culture supernatants in response to NICs formed with 3H9, a murine anti-DNA and anti-chromatin IgG (52). Healthy human neutrophils release NETs when exposed to NICs composed of purified NETs and 3H9. The NET release was attenuated in neutrophils incubated with P140, yet remained unperturbed by control ScP140 peptide, consistent with the view that NET release is regulated, in part, by autophagy. The observation that P140 drastically affects NETs release but has little or no detectable effect on mitochondria and mitophagy, highlights the importance of a NETosis pathway that would be mitochondrion-independent and modulated by the P140 peptide. The exact target of P140 in this mitochondrion-independent pathway remains to be identified. Hopefully, our future investigations will allow us to understand why P140, as possibly other molecules, apparently affects only certain forms of NETosis. This might depend on different modes of NET release, based on different stimuli and differential requirements for signaling pathways (52, 75).

Given that the P140 sequence is derived from the U1-70K ribonucleoprotein, the central component of the spliceosome and a prominent autoantigen in lupus, it is conceivable that neutrophils have developed a sensitive mechanism for detecting cellular damage and the release of nuclear contents, as may occur during NETosis. Interestingly, the phosphorylation of Ser^140^ is observed in apoptotic conditions, while the whole U1-70K protein is largely dephosphorylated by PP1-type phosphatases (76). It is therefore plausible that other modifications in the nominal sequence encompassing the RNA binding motif (present in the P140 peptide) provide cellular signals, which may fine tune the neutrophil response to cellular distress.

## Author contributions

MB performed the macroautophagy and CMA experiments. IN developed and performed the NET-IC-stimulated NET release assays. FW performed live imaging analyses by spinning disk confocal microscopy. SB performed western blotting experiments. NB and AB-S performed the experiments on isolated mitochondria. ED and JP performed the mitophagy experiments. SM and MR conceived this study, contributed the study design, and wrote the manuscript with input of all other authors.

### Conflict of interest statement

The authors declare that the research was conducted in the absence of any commercial or financial relationships that could be construed as a potential conflict of interest.
