# Supplementary material for: The Mitochondrion-lysosome Axis in Adaptive and Innate Immunity: Effect of Lupus Regulator Peptide P140 on Mitochondria Autophagy and NETosis
Source: Front Immunol. 2018 Sep 26;9:2158. doi: 10.3389/fimmu.2018.02158 (PMC6168670; doi:10.3389/fimmu.2018.02158)
Supplement: Supplementary file 1 [file Data_Sheet_1.PDF]

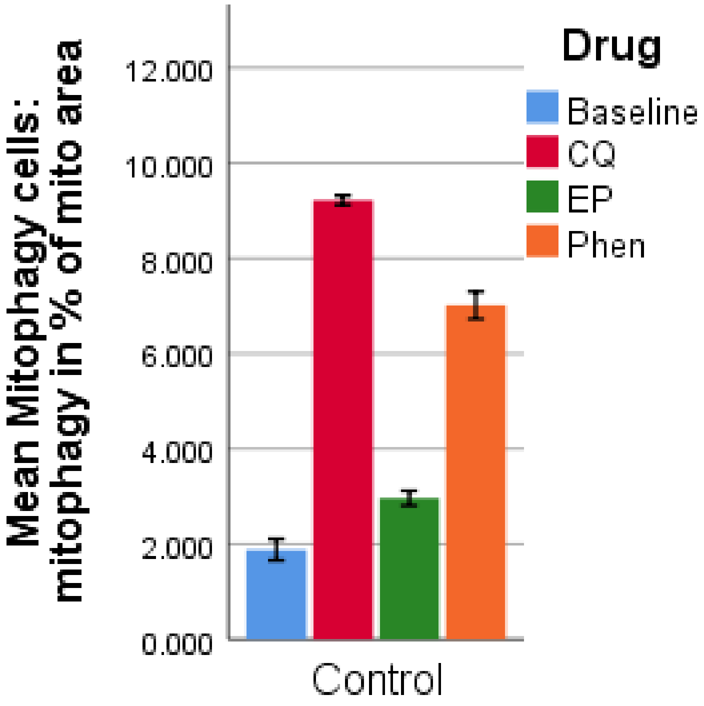

Supplementary Figure 1. Mitophagy in fibroblasts is measured as co-localisation between mitochondria and autophagomes, expressed as percentage of total mitochondrial area in fibroblasts from a healthy control. Mitophagy inhibitors chloroquine (CQ, 25 µM) and E64D with pepstatin A (EP, each 10µg/mL) increase co-localisation in this assay, by inhibiting downstream lysosomal events. Mitophagy activator phenanthroline (Phen, 20µM) increases mitophagic flux and hence increases co-localisation. Error bars are +/- standard error of the mean (SEM).

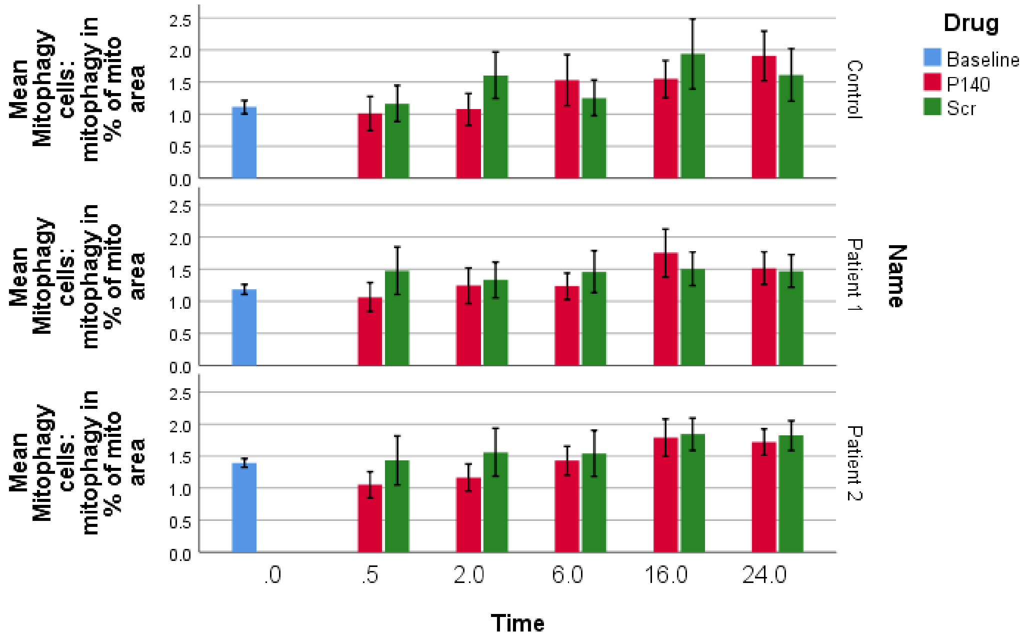

Supplementary Figure 2. Mitophagy in fibroblasts, measured as co-localisation between mitochondria and autophagomes, expressed as percentage of total mitochondrial area, was not different for P140 and ScP140 (Scr) over a 24 h-time course for each of two patients and a control. (exposure time was 0.5-24h). Results are shown as mean of three replicates. Error bars are +/- SD.

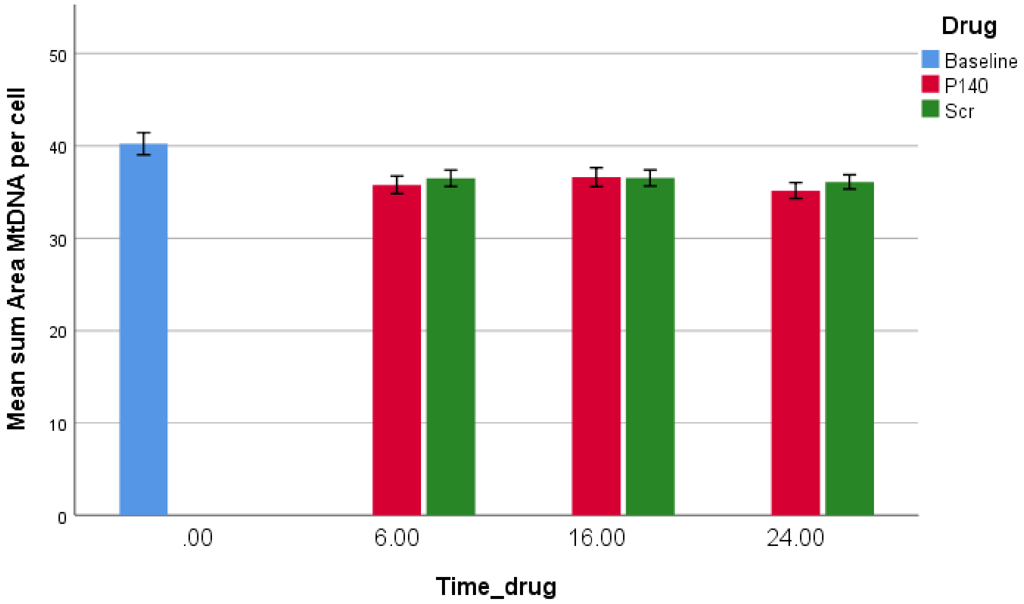

Supplementary Figure 3. Mitochondrial copy number, measured as the summed area of PicoGreen signal per cell, was not different between P140 and ScP140 (Scr). Time course expressend in hours. Error bars are +/- SEM.
